# Association of the AIRE gene with susceptibility to rheumatoid arthritis in a European population: a case control study

**DOI:** 10.1186/ar4141

**Published:** 2013-01-15

**Authors:** José-Raúl García-Lozano, Belén Torres-Agrela, Marco-Antonio Montes-Cano, Lourdes Ortiz-Fernández, Marta Conde-Jaldón, María Teruel, Alicia García, Antonio Núñez-Roldán, Javier Martín, María-Francisca González-Escribano

**Affiliations:** 1 Servicio de Inmunología, Hospital Universitario Virgen del Rocío (IBiS, CSIC, US), Avenida Manuel Siurot s/n, 41013-Sevilla, Spain; 2 Instituto de Parasitología y Biomedicina "López Neyra", CSIC, Parque Tecnológico de Ciencias de la Salud, Avenida del Conocimiento s/n, Armilla, 18100-Granada, Spain; 3 Unidad de Reumatología, Hospital Universitario Virgen del Rocío, Avenida Manuel Siurot s/n, 41013-Sevilla, Spain

## Abstract

**Introduction:**

AIRE is a transcriptional regulator playing a functional role in thymocyte education and negative selection by controlling the expression of peripheral antigens in the thymus. Recently, the *AIRE *gene was identified as a genetic risk factor for rheumatoid arthritis (RA) in genome wide association (GWA) studies performed in the Japanese population. According to the available data this association is restricted to the Asian population. However, different facts could influence the lack of association in Caucasian populations. The aim of this study was to further investigate the possible role of the *AIRE *gene in susceptibility to RA in a Caucasian population.

**Methods:**

A total of 472 Spanish Caucasian RA patients and 475 ethnically matched controls were included in the study. Three single-nucleotide polymorphisms (SNPs) (rs2776377, rs878081 and rs1055311) with a minor allele frequency >0.05 in the Caucasian population which were not included in the high-throughput platforms used in the GWA studies performed in susceptibility to RA, and two SNPs (rs2075876 and rs1800520) associated with RA in the Japanese population, were selected and genotyped using TaqMan assays.

**Results:**

No significant differences in the distribution of the alleles of rs2776377, rs2075876, rs1055311 and rs1800520 SNPs between RA patients and controls were observed. Nevertheless, the frequency of the C allele of rs878081 was significantly higher among RA patients (80.5% *vs*. 74.6% in the control group, p_c _= 0.012, OR = 1.41, 95%CI 1.13-1.75). Regarding the distribution of the rs878081 genotypes, a higher frequency of CC homozygous individuals was found in the RA patient group (65.56% *vs*. 56.47% in the control group, p_c _= 0.013, OR = 1.47, 95%CI 1.12-1.93). The *in silico *analysis predicted lower affinity to the binding-site of a motif of the transcription NF-κB family and lower transcription levels of *AIRE *gene for the rs878081C risk variant

**Conclusions:**

Our findings suggest that the *AIRE *gene is associated with susceptibility to RA in the Spanish population. Probably, this association has not been detected in the European population in the GWA studies because the earliest high-throughput platforms did not include SNP suitable markers (e.g. rs878081).

## Introduction

**Figure 1:**
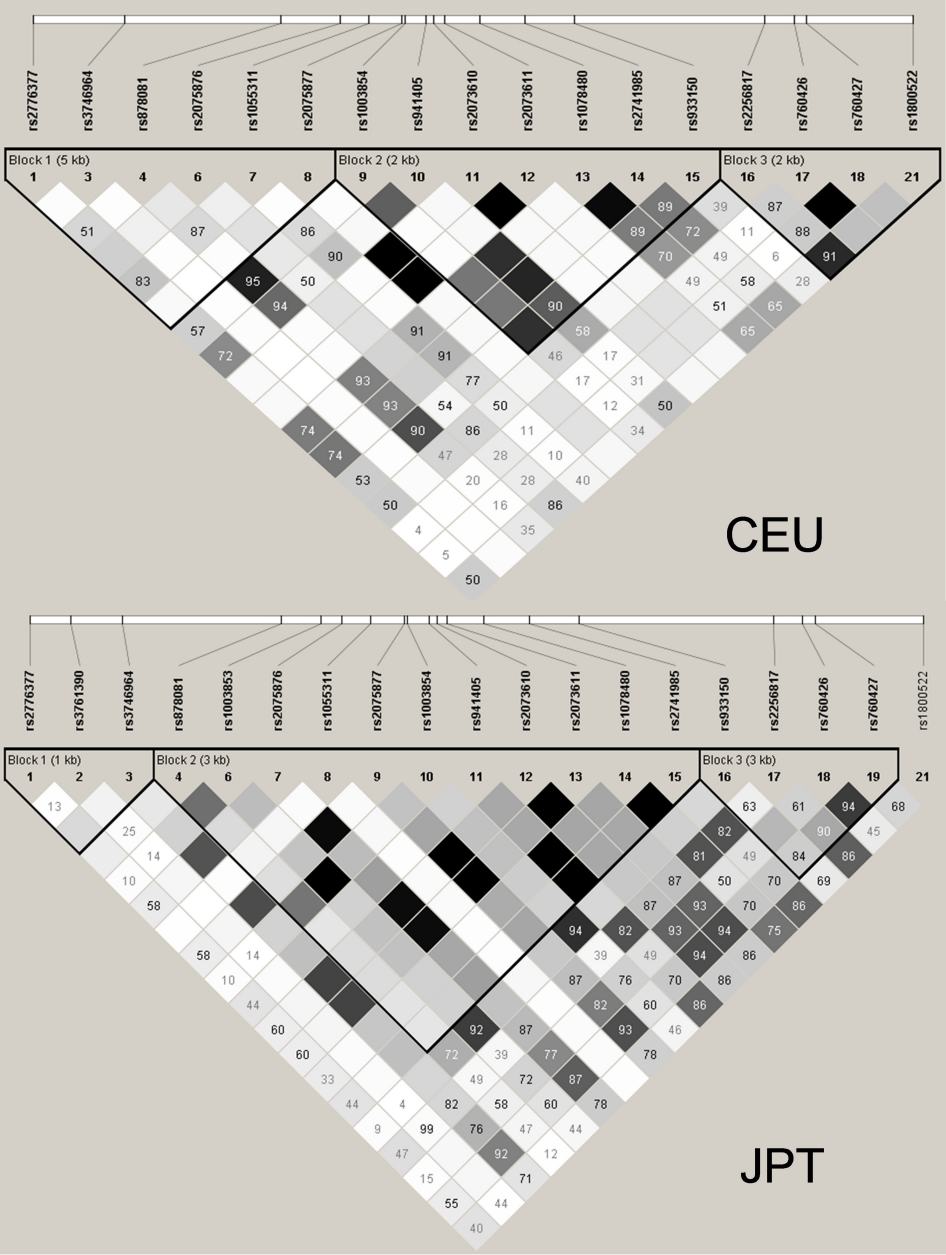
**Haplotype blocks described in the region Chr21:44529072..44541978 of the HapMap Project in the CEU and the JPT populations**. The linkage disequilibrium (LD) solid spine approach was used to define haplotype blocks. Standard color-coding was used for LD plots: white (*r*^2 ^= 0), shaded gray (0 <*r*^2 ^< 1), black (*r*^2 ^= 1). Squares without a number indicate D' = 1. CEU, Utah residents with Northern and Western European ancestry from the CEPH collection; JPT, Japanese in Tokyo, Japan.

Rheumatoid arthritis (RA) is a chronic systemic inflammatory disease that often leads to disability from joint damage and inflammation. Although RA is an uncommon disease with a worldwide prevalence of approximately 1%, this pathology has a large economic and societal cost in terms of work-related disability [1]. Both environmental and genetic factors are considered to be associated with the onset and progression of this disease. Genome-wide associations (GWA) studies have identified common genetic variations associated with numerous complex diseases [2]. Contrary to the candidate gene approach, in which a limited number of genes chosen on the basis of known or suspected biological considerations are tested, the aim of GWA studies is to check association in the whole genome without a priori hypotheses. Many gene loci have been identified as risk factors for RA in different GWA studies in European and East Asian populations. Some of these loci have been found to be restricted to a particular ethnic group but others, such as, *CCR6*, *STAT4 *and *TNFAIP3*, have been described as associated with RA in different populations [3]. Recently, the *AIRE *gene was identified as a genetic risk factor for RA in a GWA study performed in a Japanese population [4]. AIRE is a transcriptional regulator primarily expressed in medullary thymic epithelial cells, playing a functional role in thymocyte education and negative selection by controlling the expression of peripheral antigens in the thymus [5]. Therefore, *AIRE *is a good functional candidate in autoimmune diseases regardless of the population. In fact, mutations in this gene cause autoimmune polyendocrinopathy syndrome (APS1), which is one of the few known monogenic autoimmune diseases. Nevertheless, *AIRE *has not been identified as associated to RA in the European population, either in a large-scale GWA study or in a meta-analysis of GWA studies [6-10]. However, both of these studies had strong detection power, and therefore, the association of *AIRE *with RA, like that of *PAD14*, could be specific to some populations, such as in the Japanese study [4]. However, this gene has different linkage disequilibrium (LD) blocks in European and Asian populations (Figure 1), and the earliest GWA high-throughput platforms do not include any adequate tag single-nucleotide polymorphisms (SNPs) for the European population. This fact could influence the lack of association in Caucasian populations, therefore, we decided to further investigate the possible role of the *AIRE *gene in susceptibility to RA in a Spanish population.

## Materials and methods

### Study subjects

A total of 472 RA patients (351 women and 121 men) who were unrelated were included in the study. All patients meet the American College of Rheumatology (ACR) revised RA criteria [11]. The mean (SD) age of onset was 49.23 (14.8) years. Information on rheumatoid factor, anti-cyclic citrullinated peptide antibodies and Disease Activity Score in 28 joints (DAS28) for RA activity was obtained. Patients were rheumatoid factor-positive in 85.2% of the cases and anti-cyclic citrullinated peptide antibodies were present in 82.2% of them. The control population consisted of 475 ethnically matched healthy bone marrow donors who were unrelated. All the subjects were Spanish Caucasian, and they were recruited from two Southern Spanish hospitals: Hospital Universitario Virgen del Rocío (Sevilla) and Hospital Universitario Virgen de las Nieves (Granada). No significant differences in clinical features, ratio of rheumatoid factor and anti-cyclic citrullinated peptide antibodies and DAS28 score were observed between the two patient cohorts. The study was approved by all local ethical committees of the corresponding hospitals. Blood samples were obtained from subjects after they provided written informed consent, and were sent to Hospital Universitario Virgen del Rocio where the genotyping study was performed. Genomic DNA was extracted from blood leukocytes using QIAmp DNA Mini Kit (Qiagen, Hilden, Germany) according to the manufacturer's recommendations and stored at -20ºC.

### *AIRE *SNPs genotyping

An *AIRE *region (Chr21:44529072..44541978) [NCBI Reference Sequence: NC_000021.7] LD plot was generated from the Utah residents with Northern and Western European ancestry from the CEPH collection (CEU) with data obtained from HapMap (Release 28, Phase II+III, NCBI build 36 assembly, dbSNP b126). The LD solid spine approach was used to define haplotype blocks in the CEU population using the Haploview program (available at the website [12]) (Figure 1). The rs2075876, which is associated with RA in the Japanese population, was located in the LD block Chr21:44529072...44534480 encompassing a 5.4 Kb region between 5'UTR and exon 6 in the CEU population (Figure 1, CEU-block 1). A total of seven SNPs were found in the HapMap project within this block, four of them have a minor allele frequency (MAF) > 0.05 (rs2776377, rs878081, rs2075876 and rs1055311) and were the ones selected for this study (Table 1). The SNP haplotypes frequency estimation was performed using the Haploview sotfware. Additionally, rs1800520, a non-synonymous SNP (S278R), which is not included in HapMap project, was also genotyped. Genotyping of controls and RA patients was performed using TaqMan^® ^SNP Genotyping Assays (Applied Biosystems, Barcelona, Spain) in a LightCycler 480 (Roche, Barcelona, Spain).

**Table 1:** MAF of the tag SNPs included in HapMap within the Chr21:44529072

SNP	Position	MAF CEU	MAF JPT
rs2776377	44529072	0.438 (G)	0.265 (A)
rs3761390	44529667	0.000 (G)	0.023 (G)
rs3746964	44530416	0.018 (C)	0.283 (C)
rs878081	44532705	0.336 (T)	0.173 (T)
rs2075876	44533581	0.123 (A)	0.347 (A)
rs1055311	44533996	0.261 (T)	0.009 (T)
rs2075877	44534480	0.019 (A)	0.320 (A)
rs1003854	44534535	0.345 (C)	0.292 (C)
rs941405	44534838	0.422 (G)	0.341 (C)
rs2073610	44534956	0.016 (C)	0.386 (C)
rs2073611	44535109	0.016 (A)	0.386 (A)
rs1078480	44535630	0.454 (G)	0.373 (A)
rs2741985	44536295	0.460 (C)	0.341 (T)
rs933150	44537016	0.381 (A)	0.270 (A)
rs2256817	44539814	0.413 (A)	0.337 (G)
rs760426	44540242	0.159 (G)	0.359 (G)
rs760427	44540419	0.156 (A)	0.443 (A)
rs1800522	44541978	0.433 (C)	0.367 (T)

MAF: minor allele frequency; SNP: single-nucleotide polymorphism. ^a^Release 28, Phase II+III, NCBI build 36 assembly, dbSNP b126. CEU, Utah residents with Northern and Western European ancestry from the CEPH collection; JPT, Japanese in Tokyo, Japan.

### Bioinformatic analysis of *AIRE *expression

To check the effect of the variants in the *AIRE *gene expression, two different approaches were used. First, the effects of the variants on regulatory motifs were investigated using the HaploReg database [13] and quantified as: Predicted relative affinity = LOD(alt) - LOD(ref) [14], where LOD is the logarithm of the odds, ref is the reference sequence, and alt is the alternative sequence. Therefore, a negative result means that the predicted relative affinity is higher for the reference sequence whereas a positive result states that the predicted relative affinity is higher for the alternative. Second, in order to analyze the predicted expression levels, a gene-expression dataset of lymphoblastoid cell lines derived from 210 unrelated individuals from different populations of HapMap was obtained from the Gene Expression Omnibus [GEO, accession number GSE6536] database [15].

### Statistical analysis

Allele and haplotype frequency distributions were compared using the chi square test, and a corrected *P*-value (*P_c_*) was calculated from 10,000 permutations (Haploview program). *P_c_*-values < 0.05 were considered statistically significant. Odds ratios (ORs) and 95% CI were calculated according to Wolf's method using the Statcalc program (Epi Info 2002; Centers for Disease Control and Prevention, Atlanta, GA, USA). Statistical analysis of mRNA *AIRE *expression was performed by the Joncheere-Terepstra method using the SPSS sotfware (version 18).

## Results

Genotypes of rs2776377, rs878081, rs2075876, rs1055311 and rs1800520 were unequivocally assigned in 471 healthy controls and 465 RA patients. The successful rate of genotyping was > 98% for all the SNPs included, and the study population was found to be in Hardy-Weinberg equilibrium for all the polymorphisms analyzed (*P *> 0.05). Table 2 shows the frequencies of the four tag SNPs and the rs1800520 in RA patients and healthy controls. No significant differences in the distribution of the alleles of the tag SNPs, rs2776377, rs2075876 and rs1055311 were observed between RA patients and controls. Nevertheless, the frequency of the C allele of the rs878081 SNP was significantly higher among RA patients (80.5 vs. 74.6% in the control group, *P_c _*= 0.012, OR 1.41, 95% CI 1.13, 1.75) and regarding the distribution of the rs878081 genotypes (Table 3), a higher frequency of CC homozygous individuals was found in the RA patient group (65.56% vs. 56.47% in the control group, *P_c _*= 0.013, OR 1.47, 95% CI 1.12, 1.93). Alleles of the non-synonymous rs1800520 had the same frequency among patients and controls.

**Table 2:** Allelic frequencies of the SNPs studied in the AIRE in RA Spanish patients and controls

SNP (location)	Allele	Controls 2*n *= 942	Patients 2*n *= 930	*P*-value	*Pc-*value^a^	Odds ratio (95% CI)	Japanese population^b^
rs2776377 (5'UTR)	A	0.589	0.597	ns			0.265^c^
rs878081 (exon 5)	C	0.746	0.805	0.0022	0.0123	1.41 (1.13, 1.75)	0.827^c^
rs2075876 (intron 5)	A	0.114	0.116	ns			0.340^d^
rs1055311 (exon 6)	T	0.206	0.219	ns			0.012^c^
rs1800520 (exon 7)	G	0.017	0.019	ns			0.420^d^

RA, rheumatoid arthritis; SNP, single-nucleotide polymorphism, UTR: unstranlated region, ns: not significant at *P *> 0.05. ^a^*P_c_*-value, 10,000-fold permutation testing; ^b^Allelic frequencies in the Japanese population; ^c^HapMap Project, JPT (Japanese in Tokyo) population; ^d^Terao *et al*. 2011 [4].

**Table 3:** Genotype frequencies of the SNP rs878081 in RA Spanish patients and controls

Genotypes rs878081	Controls *n *= 471	Patients *n *= 465	*P*-value	*P_c-_*value^a^	Odds ratio (95% CI)
CC	0.564	0.656	0.005	0.0136	1.47 (1.12, 1.93)
CT + TT	0.436	0.344			

RA, rheumatoid arthritis; SNP, single-nucleotide polymorphism. ^a^*Pc*-value, 10,000-fold permutation testing.

In the haplotype analysis, taking into account rs2776377, rs878081, rs2075876 and rs1055311, six haplotypes with frequencies > 0.05 were identified in our population (Table 4). According to our results, the four haplotypes bearing the rs878081C allele showed a higher frequency in RA patients compared with controls, although only one of them (GCGC) reached statistical significance before permutation testing (*P *= 0.018, OR 1.38, 95% CI 1.05, 1.82). On the contrary, the two haplotypes bearing the rs878081T alleles had a lower frequency in the RA patients and one of them (GTGC) remained statistically significant after permutation testing (*P_c _*= 0.014, OR 0.67, 95% CI 0.52, 0.87). All the major haplotypes found in our population had the rs1800520C allele except the haplotype GCAC, which was found with both rs1800520 alleles (C 84% and G 16%).

**Table 4:** Allelic frequencies of the major haplotypes found in RA patients and controls

Haplotypes				Controls 2*n *= 942	Patients 2*n *= 930	*P-*value	*P_c-_*value*	Odds ratio (95% CI)
**rs2776377**	**rs878081**	**rs2075876**	**rs1055311**					
					
A	C	G	C	0.324	0.331	ns		1.03 (0.85, 1.24)
A	C	G	T	0.182	0.193	ns		1.09 (0.86, 1.38)
G	T	G	C	0.174	0.124	0.0025	0.0141	0.67 (0.52, 0.87)
G	C	G	C	0.110	0.146	0.0182	ns	1.38 (1.05, 1.82)
G	C	A	C	0.106	0.111	ns		1.05 (0.78, 1.40)
A	T	G	C	0.076	0.065	ns		0.84 (0.59, 1.20)

RA, rheumatoid arthritis; ns, not significant at *P *> 0.05. *P_c_-value, 10,000-fold permutation testing.

Case-only phenotype analysis of RA patients revealed no association between rs878081 alleles or haplotypes and mean age at diagnosis, rheumatoid factor, anti-cyclic citrullinated peptide antibodies or DAS28 score (data not shown).

**Figure 2:**
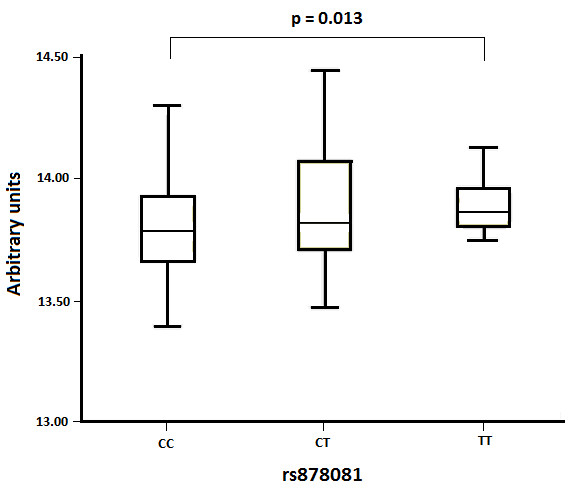
**Comparison of expression levels of AIRE obtained from the GEO database according to the genotype of rs878081**. Results of AIRE mRNA expression are shown in a boxplot. The *P*-value was obtained by the Joncheere-Terepstra method.

The *in silico *study using the HaploReg database [14], predicts a motif binding site that spans the rs878081 location for transcription factors of the nuclear factor-kappa B (NF-κB family [16] The difference between the LOD score of the T allele and the LOD score of the C allele was +2 (positive), therefore, this model predicts alterations of the affinity to regulatory motifs, which is higher for the variant T than for the C allele. Additionally, when the *AIRE *expression levels were analyzed using the expression profiles from the GEO database [15], a statistically significant difference in the mean levels of expression of the rs878081 alleles was found (*P *= 0.013). The transcription of *AIRE *was decreased by the C allele compared with the T allele (Figure 2).

## Discussion

Replication studies in different populations have an important role in the validation of common genetic variation associated to complex diseases, strengthening the confidence of initial association reports. In the present work in a Spanish population we have replicated the association between the *AIRE *gene and RA that was recently identified in the Japanese population [4].

In the GWA study performed in the Japanese population, two SNPs located in the *AIRE *gene region, rs2075876 and rs760426, were described to be associated with RA. Nevertheless, association with this gene had not been found in European populations in previous GWA studies [6-10]. Failure to identify the association in European populations could be explained by several reasons. Some of these reasons may be based on differences between European and Asian populations in the polymorphism of this gene. According to HapMap data, the region Chr21:44529072..44541978 included 18 tag SNPs, of which 15 have an MAF > 0.20 in Japanese in Tokyo, Japan (JPT) vs. 10 in CEU (Table 1). Additionally, four of these tag SNPs with an MAF > 0.20 in JPT have an MAF < 0.05 in CEU, whereas in contrast, MAF > 0.20 in CEU and MAF < 0.05 is found only in one case. Specifically, the SNPs identified as risk markers in the Japanese population, have a much lower MAF in the Caucasian populations included in the HapMap project (rs2075876A 0.123 in CEU and 0.096 in Tuscan in Italy (TSI) and rs760426G in 0.159 CEU and 0.163 in TSI) than in the JPT (0.347 and 0.359, respectively) and other Asian populations (0.437 and 0.389 in Han Chinese in Beijing, China (CHB) and 0.417 and 0.413 in Chinese in Metropolitan Denver, Colorado (CHD) respectively) included in this project. Furthermore, the LD blocks in the *AIRE *region locus are different in Caucasian and Asian populations (Figure 1). The *AIRE *region is not included in GWA high-throughput platforms of Affimetrix^® ^used in several RA susceptibility studies [6]. Additionally, the earliest GWA high-throughput platforms of Illumina^® ^used in RA susceptibility studies do not include any tag SNPs with an MAF higher than 0.2 for the European population within the LD block where the rs2075876 is located (the LD block between 5'UTR and exon 6 of the *AIRE *region) [7,9]. For these reasons, we focused our study on the LD block encompassing a 5.4 Kb around of 5' *AIRE *region locus. The rs878081 SNP was found to be associated to RA in our population, even with a higher OR (1.41) than the repoted in the Japanese population for the rs2075876 (OR = 1.18). Therefore, according to our results, some genetic variants with associations to disease that have been described to be restricted to a specific ethnic group could lack this association in other populations, because the tag SNPs of some genome regions included in GWA platforms are more or less suitable according to the population. This fact could also explain why some associations found in candidate gene studies may not be replicated in GWA studies, due to suboptimal representation and coverage of the risk variants with the tag SNPs used in the high-throughput platforms [17,18].

The association study performed with haplotypes showed that the susceptibility is strongly influenced by the rs878081 SNP, because all those haplotypes bearing the rs878081C allele showed a higher frequency in RA patients compared with controls. Despite the different distribution of haplotypes among European and Asian populations, the risk allele rs2075876A in the Japanese population is in LD with the risk allele rs878081C in the Spanish population. The rs878081 is located in exon 5 but it is a synonymous polymorphism, and therefore, it does not introduce functional alterations in the AIRE protein; hence, other non-synonymous SNPs could contribute to the disease. The HaploReg database predicts a lower affinity of the factor-binding site to the motif of transcription of the NF-κB family for the rs878081C risk allele [14,16]. Additionally, results of the *in silico *analysis of the dataset of lymphoblastoid cell lines of GEO showed that similarl to the rs2075876A risk allele [4], transcription levels of *AIRE *are lower for the rs878081C risk allele [15]. It has been demonstrated that NF-κB2 is required for the transcriptional regulation of the *AIRE *gene in the thymus, contributing to the maintenance of central tolerance in an AIRE-dependent manner [19]. According to our results, the lower affinity to the C allele for the transcription factor motif could be the cause of the lower transcription levels found in the *in silico *analysis. These lower levels of transcription of the gene could promote failure of negative selection in the thymus, and as a consequence, increase the survival of autoreactive T cell clones.

We also checked the association of the non-synonymous SNP, rs1800520 (S278R) located in the exon 7, which was found to be associated to RA in the Japanese population. Nevertheless, no differences in allele frequencies of this SNP were found when comparing patients and controls in our population. The MAF of the rs1800520 in the Spanish population was 0.017 vs. 0.420 in the Japanese population [4]. This is probably the cause of the discrepancies observed in the association with rs1800520 in both populations. Finally, in our study we did not include the rs760426, which was also found to be associated with RA in the Japanese population, because the MAF of this SNP in the CEU population is < 0.2 and it is located in another LD block.

## Conclusions

In summary, this is the first study establishing a relationship between the *AIRE *gene and the susceptibility to RA in a European population. This relationship could not have been detected in the GWA studies because of the differences in both the frequency of SNPs and in the structure of the linkage blocks between the different ethnic groups that would determine better or worse adequacy of the SNP markers included in the platforms.

## Abbreviations

ACR: American College of Rheumatology; APS1: autoimmune polyendocrinopathy syndrome; CEU: Utah residents with Northern and Western European ancestry from the CEPH collection; CHB: Han Chinese in Beijing, China; CHD: Chinese in Metropolitan Denver, Colorado; DAS28: Disease Activity Score in 28 joints; GEO: Gene Expression Omnibus; GWA: genome-wide association; JPT: Japanese in Tokyo, Japan; LD: linkage disequilibrium; MAF: minor allele frequency; OR: odds ratio; RA: arthritis rheumatoid; SNP: single-nucleotide polymorphism; TSI: Tuscan in Italy.

## Competing interests

The authors declare that they have no competing interests.

## Authors' contributions

JRGL, MAMC, ANR and MFGE contributed to the conception and design of the research, analysis and interpretation of the data, and drafting and revision of the manuscript. BTA, LOF and MCA performed the experiments and analyzed the data. MT, AG and JM contributed samples and patient information. All authors read and approved the final version.
